# Rectal Stricture

**Published:** 1897-08

**Authors:** 


### Rectal Stricture.

There has been quite an acrimonious discussion going on between two very eminent men in rectal diseases, Dr. J. M. Mathews, of our city, and Dr. C. B. Kelsey, of New York, both able in their specialties, both concise and instructive writers, both men of great ability, and both publishers of excellent works on rectal diseases, the great question at issue being stricture of the rectum. Mathews maintains that syphilis and cancer are the only causes of stricture of the rectum; Kelsey, on the other hand, claims that there are other causes besides syphilis and cancer which produce stricture.

I am nothing but a drop, as it were, so far as the treatment of rectal diseases are concerned, compared with these two great lights, yet I have done enough rectal treatment, and seen enough cases of rectal stricture, to form an opinion as to the cause of rectal stricture. I will have to take issue with Dr. Mathews in regard to rectal strictures, for I am satisfied I have seen and treated a good many cases of stricture of the rectum where there was no cancer present, and no history could be made out of inherited or acquired syphilis.

Mr. J. H., a carpenter, had, three years ago, a severe case of dysentery. When he applied to me for treatment he had stricture of the rectum. He dated his trouble from the time of the dysentery. I could find no cancerous condition, and could trace no syphilis. He recovered. I am satisfied that the dysentery caused the stricture.

Mrs. W. A. S., a strong, rugged woman, applied to me with stricture of the rectum. Two years prior to that time she had a severe attack of dysentery. No cancer present, and no history of syphilis. She recovered.

Miss D. T., nineteen years of age, had pronounced stricture.

No cancer and no syphilis discovered. Had had dysentery one year before.

Mrs. B. P., applied with a well-defined case of stricture of the rectum. Stricture commenced shortly after the birth of her first child. She was delivered with forceps, I judge. She said some kind of instruments were used in her delivery. She was in bed ten weeks after the delivery of her child, and had had two operations performed. Found decided stricture of rectum, as though resulting from some cicatrice from some sort of an operation. There was no cancer and no syphilis, I honestly believe. She was very ignorant, yet there were no syphilitic indications which she described. I believe she never had had syphilis.

James P., a boy seven years old, applied for relief. Some time before his mother consulted me. Her boy had fallen upon a board fence. What was the nature of the injury I could hardly tell. Evidently there was contusion, though no result of any wound could I make out. What caused his mother's attention to the case was the size of the feces. They resembled an earth worm—about the size of an earth worm, and covered with mucus. I could not possibly introduce my forefinger into the rectum. I did pass a No. 22 English sound into rectum. I gave the boy chloroform and stretched the sphincter. Gave his mother a rectal bougie to be passed every day. The boy apparently is well. I am satisfied there is no cancer in his case, and am also satisfied there is no syphilis.

I have other cases upon my note-book, yet I think these few cases are sufficient to prove that we may have stricture of the rectum from other causes besides cancer and syphilis. I am willing to admit that syphilis and cancer are the most common and frequent causes of stricture of the rectum, but that they are the only causes I am a long way from admitting. Why Dr. Mathews, a man as skillful as he, and a man who has seen so many cases of stricture of the rectum as he has, should claim it surprises me. I am just as positive that stricture of the rectum is occasionally caused by benign means as I am that I am a living being. I think any one who has seen many cases of stricture of the rectum must come to this conclusion. I dislike to antagonize as prominent a rectal specialist as is Dr. Mathews, yet it seems to me to be my duty to tell the truth. I do believe that there are a number of other causes for stricture of the rectum besides cancer and syphilis. When we do have stricture of the rectum from cancer, our patient will die inside of two or three years; when we do have stricture of the rectum that cant be cured without the use of anti-syphilitics, we become quite well satisfied that the stricture is not a syphilitic stricture. I have had several strictures which I have cured without the use of medicine. Is this not a pretty positive indication that the stricture is not syphilitic?—*Dr. Monroe in Lancet-Clinic.*

